# What is pregnancy and what is disease? a critique of Smajdor and Räsänen’s “is pregnancy a disease? a normative approach”

**DOI:** 10.1007/s40592-025-00271-0

**Published:** 2025-11-03

**Authors:** Maja Sidzińska

**Affiliations:** 1 University of Pennsylvania, Philadelphia, United States; 2 Rowan University, Glassboro, United States

**Keywords:** Pregnancy, Disease, Classification, Definition, Risk, Social determinants of health

## Abstract

Anna Smajdor and Joona Räsänen argue that pregnancy should be classified as a disease (In: Is Pregnancy a Disease? A Normative Approach, vol 51, 2025). But their argument faces a problem not yet raised by other critics (see In: Pregnancy, Pain and Pathology: A Reply to Smajdor and Räsänen, vol 51, 2025; In: No, Pregnancy Is Not a Disease, vol 51, 2025; In: Failing to Deliver: Why Pregnancy Is Not a Disease, vol 51, 2025). To classify a phenomenon, e.g., pregnancy, as belonging to a category, e.g., disease, one must characterize the category as well as the phenomenon. But Smajdor and Räsänen do neither. Indeed, they reject every apparent candidate theory of disease, and they do not define pregnancy. The closest they come to characterizing disease’ is positing the *converse* of Quill Kukla’s criteria for non-disease (In: Infertility, Epistemic Risk, and Disease Definitions, vol 196, 2019). The converse of Kukla’s criteria are: (1) there is agreement and consistency between definitions, (2) a unified physical syndrome is present, (3) the risks are medical rather than social, and (4) the risks are not only ‘risks’ within a particular set of social values (In: Is Pregnancy a Disease? A Normative Approach, vol 51, 2025). Smajdor and Räsänen then claim that pregnancy meets these criteria. But contra what they argue, I show that pregnancy fails to meet these criteria, or that, at best, whether or not pregnancy meets some of them is not established. If Smajdor and Räsänen abandon even those suggested criteria, however, they have no basis for classifying pregnancy—whatever it is—in any way at all. On these grounds, I contend they have not shown that pregnancy should be classified as a disease.

## Introduction

Anna Smajdor and Joona Räsänen argue that pregnancy should be classified as a disease (2025). Alternatively, we can interpret their thesis to be merely that there are pragmatic reasons to classify pregnancy as a disease (see 2025, 37). Either way, their argument faces a problem not yet raised by other critics (see Baron 2025; Colgrove and Rodger 2025; Rezkalla and Smith 2025). To classify a phenomenon, e.g., pregnancy, as belonging to a category, e.g., disease, one must characterize the category as well as the phenomenon. But Smajdor and Räsänen do neither. Indeed, they reject every apparent candidate theory of disease, and they do not define pregnancy. The closest they come to characterizing disease is positing the *converse* of Quill Kukla’s criteria for non-disease (2019).^1^ The converse of Kukla’s criteria are: (1) there is agreement and consistency between definitions, (2) a unified physical syndrome is present, (3) the risks are medical rather than social, and (4) the risks are not only ‘risks’ within a particular set of social values (Smajdor and Räsänen 2025, 42). They then claim that pregnancy meets these criteria. But contra what they argue, I show that pregnancy fails to meet these criteria, or that, at best, whether or not pregnancy meets some of them is not established. If they abandon even those suggested criteria, however, they have no basis for classifying pregnancy—whatever it is—in any way at all. On these grounds, I contend Smajdor’s and Räsänen’s argument does not succeed on its own criteria, and they have not shown that pregnancy should be classified as a disease.

## Smajdor and Räsänen’s argument

Smajdor and Räsänen’s argument to the effect that pregnancy should be classified as a disease takes up a number of disparate issues in its defense. The argument begins by focusing on similarities that pregnancy shows with disease: its symptoms and risks, including the risk of maternal mortality, as well as the fact that it warrants medical attention (2025, 37–38). They compare pregnancy to measles—a paradigmatic example of disease—and aim to show that the comparison suggests that pregnancy is more clearly a disease than is measles on account of its prevalence, risks, and harms.^2^

Smajdor and Räsänen then consider several theories of disease. They take Rachel Cooper’s (2002) theory of disease as an example of a subjectivist view of disease. On Cooper’s view, a phenomenon is a disease if (1) it is bad for the person who suffers from it, (2) the sufferer is unlucky to suffer from it, and (3) it can be treated medically. Smajdor and Räsänen propose that pregnancy may meet the first two criteria, and that it definitely meets the third criterion. They note, however, that we should “think carefully about the social conditions that inform a patient’s perception of [a phenomenon], before endorsing too eagerly the idea that subjective value is what makes [the phenomenon] a disease or not” (2025, 39). They claim this to guard against the possibility that the frequent subjective valuation of pregnancy as good or wanted disqualifies it from counting as a disease. The social condition they think may bias such valuation is the prevalence of pronatalist values.

Next Smajdor and Räsänen turn to descriptive theories of disease, under which a phenomenon counts as disease when it is treated as such by the medical community (2025, 39; see also Merskey 1986). On such a theory, they argue, pregnancy cannot be easily classified as a disease because, while pregnancy is usually associated with medical treatment, infertility—construed as the absence of pregnancy—is also often treated medically. On this theory, they note, pregnancy turns out to be “…both a disease and not a disease!” (2025, 40). They drop this theory of disease on this and other grounds.

Smajdor and Räsänen then consider dysfunction theories of disease. They note that while “[m]easles is dysfunctional… it is commonly regarded as a mark of a healthy body that it can become pregnant” (2025, 40). In other words, pregnancy is seen as a function, so it is not a disease on the dysfunction theory of disease. While pregnancy may carry a risk of harm, it is unlike measles in that it is not a dysfunction. But Smajdor and Räsänen criticize dysfunction theories of disease on the grounds that the notion of function is fundamentally teleological and therefore normative: it presupposes that a body should behave in a particular way. But how a body should behave, they claim, cannot be observed in nature, that is, it cannot be objectively established. It requires a value judgment over and above whatever may be observed. The question arises: whose, or which, value judgment? There appears to be no objective answer, thus Smajdor and Räsänen reject dysfunction theories of disease on which pregnancy would not count as such.

Smajdor and Räsänen then look to ‘normal species function’ accounts against which dysfunction may be defined (2025, 41). Such accounts define normal function as that which is statistically common relative to a given reference class. Implicitly, then, what is not statistically common is a dysfunction, and may count as disease. While Smajdor and Räsänen do not claim on such grounds that pregnancy is a disease, they do use the idea of normal species function to challenge the view that pregnancy is a normal function, since most people are not pregnant most of the time.^3^

Naturally, they then consider Christopher Boorse’s biostatistical theory of disease, which is ostensibly a value-free theory of disease.^4^ According to biostatistical theory, diseases are states that not statistically typical within certain reference classes (see Boorse 1977). Since pregnancy contributes to reproduction, and reproduction is a normal function for Boorse (1077, 555ff), it would not count as a disease. Other factors complicate the picture: one, that on such a view, some phenomena would count as disease which we do not wish to count as such (e.g., homosexuality), and two, that what contributes to survival or reproduction is environment-relative, and therefore unstable (Smajdor and Räsänen 2025, 41–42). In this case, depending on whether full ectogenesis is developed and used widely as an alternative to pregnancy, for instance, pregnancy might either be considered a disease or not a disease.^5^ But this is counterintuitive. Smajdor and Räsänen note that other unwanted consequences of biostatistical theory result from complications having to do with evolutionary tradeoffs *between* survival and reproduction (2025, 41–42). They do not consider the theory further.

Ultimately, Smajdor and Räsänen do not find any of these theories of disease to settle the matter. They suggest that these theories either do not return results on the question of pregnancy that account for select features of it such as its negative symptoms, or that they are plagued by independent problems.

### Smajdor and Räsänen’s reformulation of Kukla’s criteria

#### Kukla’s argument

Having abandoned the above candidate theories of disease, Smajdor and Räsänen turn to Kukla’s argument in “Infertility, Epistemic Risk, and Disease Definitions” (2019). Kukla argues that the definitions of various biomedical phenomena that drive disease classification are themselves value-laden. Depending on how, for example, mercury poisoning—as an independent variable, is defined, certain dependent variables either will or will not be found to cause it or to be associated with it. Demarcating biological phenomena is required, however, if any research or clinical practice is to occur.^6^ Furthermore, whatever its benchmark, Kukla argues that insofar as whether or not a phenomenon is affected by certain dependent variables is to be determined inductively, such a determination is itself value-laden given that our *p*-values are decreed in advance as matters of evaluative judgment (2019, 4411). Kukla’s interest in these issues occurs in the context of infertility, in particular with regard to questions about whether or not it should be conceptualized as a disease. These questions are motivated by the fact that social values influence infertility’s classification, which in turn influences its medical treatment—or lack thereof.

Kukla provides “several *prima facie* reasons” to challenge the classification of infertility as a disease (2019, 4421). These are:

(1) The deep conflict between the different definitions, even including inconsistencies concerning what sorts of entities can have infertility; (2) The lack of any kind of unified physical syndrome; and (3) The fact that the risks of infertility seem to be social rather than medical (and that these are only clearly ‘risks’ given a certain ideologically infused set of expectations and values). (Kukla 2019, 4421)

With regard to (1—conflict and inconsistency between definitions, Kukla demonstrates the variety of ways infertility is defined, even by authoritative sources (2019, 4415ff). They show that collectively, these definitions are incoherent and contradictory: in the end, we do not know what they are definitions *of*. With regard to (2), Kukla notes that infertility refers to a diversity of phenomena—phenomena which are marked by many distinct etiologies. So ‘infertility’ is an umbrella term, designed to capture a wide variety of biological phenomena. With regard to (3), Kukla notes that understandings of such processes as disease—something to be treated—may only make sense as dysfunctions given value-laden assumptions about how reproduction should occur. So, Kukla argues, not only is infertility an umbrella term that cannot be consistently operationalized in biomedical practice, but it is ideologically driven by the idea that one ought to produce genetically related descendants, rather than, for instance, adopt children.

## Smajdor and Räsänen on Kukla

As Smajdor and Räsänen have it, Kukla criticizes the idea that infertility is a disease because there is

1. Conflict and inconsistency between definitions,
2. Lack of any unified physical syndrome,
3. The risks are social rather than medical,
4. The risks are only ‘risks’ within a particular set of social values. (2025, 42)

Smajdor and Räsänen borrow these reasons and “apply these to pregnancy in reverse” (2025, 42). Thus the *converse* of Kukla’s reasons for avoiding classifying infertility as a disease are reasons *for* classifying pregnancy as a disease for Smajdor and Räsänen. We can formulate these reasons as follows and take them to mean that pregnancy is a disease if

1. There is agreement and consistency between definitions,
2. A unified physical syndrome is present,
3. It is not the case that the risks are social rather than medical,
4. It is not the case that the risks are only ‘risks’ within a particular set of social values.

These reasons can be understood as suggested markers of disease. Smajdor and Räsänen put the point as follows: “phenomena that perform well on these criteria are more convincingly to be understood as diseases” (2025, 42). They claim that pregnancy meets the first criterion because “one cannot be ‘a little bit’ pregnant” (2025, 42). This is taken to mean that no one is confused about the definition of pregnancy. They suggest that pregnancy meets the second criterion because whether or not one is pregnant is a binary matter (2025, 42). Furthermore, they claim, pregnancy is objectively verifiable, rather than relative to how one feels (2025, 42). The implication is supposed to be that in pregnancy, a unified physical “syndrome” is present. (Henceforth, however, I will not refer to any ‘syndrome,’ but rather to a ‘phenomenon’, because while ‘phenomenon’ is neutral, ‘syndrome’ intimates disease, biasing the framing of the central question. A *phenomenon* can be understood generally as an experienced event. A neutral term, such as ‘phenomenon’, is required given that whether pregnancy is harmful or otherwise problematic is the very thing in question.) With regard to the third criterion, Smajdor and Räsänen claim that the risks are primarily medical, although they acknowledge that they can be both medical and social (2025, 42). And when it comes to the fourth criterion, Smajdor and Räsänen write that given Kukla’s argument that “…it is the social norm that people should have children that makes infertility a disease… [W]e would suggest it is the social norm that people should have children that tends to preclude our ability to recognise pregnancy as a disease” (2025, 42).

It may be that Smajdor and Räsänen ought not reject the theories of disease discussed in Section 2 (see Colgrove and Rodger 2025; Rezkalla and Smith 2025) and ought not adopt Kukla’s view without more elaboration. I set these issues aside and argue that *even if* we grant Smajdor and Räsänen these priors, their argument does not go through.

## Pregnancy does not meet Smajdor and Räsänen’s criteria

In what follows, I show that pregnancy fails to count as disease on the criteria Smajdor and Räsänen adopt, or at the least, that it has not been established that pregnancy meets the criteria.

### Criterion 1: Agreed-upon, consistent definition

Consider the first criterion: that there is agreement and consistency between definitions of pregnancy. This is demonstrably false. Pregnancy is defined in a number of different and conflicting ways, some of which are enumerated below. Each candidate definition is criticized (much as Kukla criticizes definitions of infertility (see 2019, 4415ff)).

This set of definitions is drawn primarily from some of the top-most results of basic internet searches for “medical definition of pregnancy” and “biological definition of pregnancy.”^7^ Both searches included authoritative and widely-referenced governmental sources, and there was some overlap in the search results across these searches. The search for “biological definition of pregnancy” returned commonly used definitions in biological and medical reference texts that are relevant for medicine and discussions of pregnancy in medical terms such as Smajdor and Räsänen’s. They are also relevant given Smajdor and Räsänen’s references to evolution. In addition to the definitions generated by the searches, I included John Avise’s definition, given that he explicitly discusses complications with pregnancy definition.^8^ The goal was to highlight accessible yet medically-relevant and rigorous definitions—the kind of definitions that bioethical scholarship may plausibly rely on. As will become evident, the conflict in definitions does not occur across obscure or highly specialized contexts, but rather in commonly used yet authoritative publications. Even if the presented definitions were discarded, and seven alternatives selected, the same or similar problems to those I outline below would be highly likely to occur.

1. The United States National Library of Medicine says that “[t]he first week of pregnancy starts with the first day of a woman’s menstrual period. She is not yet pregnant” (“Fetal Development,” n.d.). This is contradictory, since it is impossible both that pregnancy has begun and that someone is not yet pregnant. This definition is a result of the fact that medical practitioners generally date human pregnancy based on the timing of menstruation, but this does not state what the phenomenon is that is being dated. It also excludes non-human pregnancy; more is said about this below.
2. The United States Department of Health and Human Services defines pregnancy as “the period in which a fetus develops inside a woman’s womb or uterus” (Eunice Kennedy Shriver National Institute of Child Health and Human Development 2017). Given this definition, any period of time in which a fetus is developing within a uterus may count as pregnancy. But this is occurring *all* the time in *some* women; in other words, this definition says nothing about the specific physiological relationship between a particular fetus to a particular uterus, which involves implantation, and an anatomical relationship in the form of a placenta and umbilical cord. The next problem is that not only human beings undergo pregnancy. This issue may be forgiven given the definition’s context: human health, but it should not be overlooked given that Smajdor and Räsänen appeal to evolutionary considerations when they argue against dysfunction theories of disease (2025, 41, 42). Furthermore, it is relevant to the biomedical context that not only human beings undergo pregnancy, since, for better or worse, mice and other non-human animals are used to model pregnancy in biomedical research (Lim and Wang 2010; Carter 2020).
3. BiologyOnline gives yet another definition of pregnancy: there, pregnancy is defined as “[t]he condition of having a developing embryo or foetus in the body, after union of an ovum and spermatozoon” (Biology Online 2019). This too fails to specify the relationship of the gestating organism to the fetus. It may describe adelphophagy, which occurs when fetuses eat sibling embryos during gestation—a behavior that occurs in lamnoid sharks, for instance. It may also include the brooding process of the Australian gastric-brooding frog *Rheobatrachus silus*, in which a female frog ingests her fertilized eggs for purposes of internal incubation (Corben et al. 1974; Tyler and Carter 1981).
4. The online Britannica encyclopedia defines pregnancy as a “process and series of changes that take place in a woman’s organs and tissues as a result of a developing fetus” (“Pregnancy | Britannica” n.d.). This also fails to specify the relationship between the woman and the fetus; the fetus is implicitly understood as *any* developing fetus that has an unspecified physiological effect on any woman.
5. OpenText says pregnancy “begins with the fertilization of an egg and continues through to the birth of the individual” (Molnar and Gair 2015). But fertilization may occur outside of an organism’s body, for instance, in a petri dish as in the process of in vitro fertilization (IVF), or in the open environment such as in the process of broadcast spawning (a form of reproduction involving the release of eggs and sperm into the open environment, usually the water column). Fertilization in these cases never leads to birth, where birth is understood as parturition, at least not directly. Either the embryo generated by IVF must first be implanted into a uterus, or the process may lead rather to *hatching*, not birth.^9^ Furthermore, this definition also fails to specify the relation between the particular gestating organism and the particular embryo. Finally, it is not the case that birth must occur after pregnancy; spontaneous or elective abortion, or resorption, may occur, and these do not retroactively indicate an absence of pregnancy.
6. For Zoey Pascual and Michelle Langaker, pregnancy is “a state of having implanted products of conception located either in the uterus or elsewhere in the body” (2022). This is the definition I accept. Nevertheless, implantation is a gradual process, and includes three phases as follows: *apposition*, *attachment* (adhesion), and *penetration* (“invasion”) of the embryo to the uterus (Guzeloglu-Kayisli et al. 2007). Apposition is the unstable attachment of the embryo to the endometrial surface; attachment is the stronger adhesion of the embryo; and penetration is the invasion of the embryo through the luminal epithelium that is deep enough to establish a vascular relationship with the mother (Guzeloglu-Kayisli et al. 2007; Wooding and Burton 2008). Further vascular integration later occurs in which the maternal spiral arteries are restructured to enhance blood flow between the fetus and the mother (Kim and Kim 2017, 354). This occurs with the development of the placenta and umbilical cord (see Heil and Bordoni 2022).
7. John Avise notes that “[c]onventionally, pregnancy is defined as a period of internal gestation that precedes the birth of free-living progeny” (2013, 5). He questions what should count as pregnancy from an evolutionary point of view (Avise 2013; Chap. 1). His definition does not specify in much detail the kind of gestation in question (and it is teleological since it presumes birth as an outcome). Citing Wourms (1981) and Blackburn (1994; 2000), Avise claims that gestational phenomena can be “matters of degree,” which “defy attempts to define pregnancy by any single universal criterion” (2013, 15ff). Avise opts to “[adopt] a heuristic approach by comparing a wide variety of gestational phenomena” in his book about the evolution of pregnancy (2013, 17). He claims that “it is much more important to appreciate the diversity of nature’s incubational modes than it is to quibble about formal definitions of pregnancy” (Avise 2013, 15).

Given these different and conflicting definitions, it is clear that pregnancy does not meet Smajdor and Räsänen’s first criterion for disease. Even if we limit the definition to the human context given our presumed interest in what counts as human disease rather than veterinary disease or biological pathology, definitions (1), (2), (4), and (6) still differ and conflict.

### Criterion 2: unified phenomenon

These conflicting definitions immediately raise the question of what the phenomenon is that the term ‘pregnancy’ aims to refer to; that is, these definitions raise the question of whether pregnancy meets Smajdor and Räsänen’s second criterion for disease.

Jointly, the definitions of pregnancy do not refer to any unified phenomenon, as demonstrated above. Jointly, the definitions pick out the phenomena of (1) being pregnant and not being pregnant, (2) a period of time during which some fetus develops inside of some womb, (3) adelphophagy, (4) a series of changes in a woman’s body (caused by some fetus), (5), fertilized eggs in water columns and in petri dishes, (6) the implanted products of conception in a uterus, and (7) various gestational phenomena, including offspring in marsupia or brood pouches. Thus, when we consider the definitions jointly, pregnancy does not meet Smajdor and Räsänen’s second criterion for disease, because the phenomena these definitions refer to are quite diverse (setting aside that it is not clear what is being referred to at all in some cases).

But candidate definitions of pregnancy may *individually* refer to unified phenomena. Some of these definitions do, although this still does not settle what target system—what referent—we are aiming to select by the term ‘pregnancy.’

We may begin by considering definition #1. Definition #1 does not refer to any unified phenomenon. That’s because on this definition, pregnancy has started when someone is not yet pregnant, thus referring to everything or nothing at all.

If we select definition #2, then strictly speaking, pregnancy is occurring all the time, yet according to this definition we are not able to say in whom it is occurring, given the problems mentioned above. In this case, while a unified phenomenon may exist, we cannot identify any particular instances of it.

If we select definition #3, then we must include adelphophagy as pregnancy, as well as any ingested fertilized eggs. If you had such eggs for breakfast, you may just be pregnant.

If we select definition #4, we must admit changes in a woman’s organs or tissues that are caused by any fetus, not necessarily the one that is implanted in her uterus. (While it is easy to intuitively fill in the conceptual gap left by this definition and assume that the fetus in question is the implanted one, I suggest we need a greater degree of specificity from a definition.)

On definition #5, all fertilized eggs count as instances of pregnancy, including those in petri dishes, cryopreservation straws, birds’ nests, or the open environment. This appears to be a unified phenomenon.

If we select definition #6, and take pregnancy to be “a state of having implanted products of conception located either in the uterus or elsewhere in the body,” it appears we are picking out a unified phenomenon as well.

If we select definition #7, and use ‘pregnancy’ to refer to all evolved gestative phenomena, including potentially technological ones such as ectogestation, we are also referring to a unified phenomenon.

But which definition picks out the phenomenon of pregnancy? Smajdor and Räsänen do not say. I suspect, however, that they view the phenomenon of pregnancy to be the one picked out by Pascual and Langaker’s definition: a state of having implanted products of conception located either in the uterus or elsewhere in the body. On Pascual and Langaker’s definition, embryonic implantation is the marker of pregnancy. ‘Implanted embryos’ may be a unified enough physiological phenomenon. Even so, this definition is not without complications. First, as noted above, implantation is a gradual process. Is there a moment when an embryo is implanted enough that we wish to select as meeting some benchmark for pregnancy? Smajdor and Räsänen refer to the joke that “one cannot be ‘a little bit’ pregnant” and they claim that pregnancy “is binary… one either is pregnant or not” (2025, 42), but it appears that, strictly speaking, this is not the case. Let us set this problem aside, however, as boundary problems are ubiquitous when it comes to questions of classification. We can claim, at least, that there are cases when it is clear that embryonic implantation either has or has not occurred.

Smajdor and Räsänen continue, however: “Moreover, pregnancy is objectively verifiable…” (2025, 42). This is rather true, but also not without caveats. There are two ways of measuring (human) pregnancy—urine tests and blood tests, both of which aim to detect human chorionic gonadotropin (HCG) (Pregnancy Test 2022). HCG is a hormone required for biochemical changes in the uterus that enable implantation (Betz and Fane 2024; Kim and Kim 2017). Urine tests are accurate, correctly detecting pregnancy if performed a sufficient amount of time after implantation, in 97–99% of cases; blood tests are even more accurate. False negatives can occur, however, if the test is administered before a high enough level of HCG has accumulated in the pregnant body, especially for urine tests. False positives are rare, but may occur in cases of so-called “missed miscarriage,” when the embryo or fetus has ceased developing but remains implanted. (Notably, however, under Pascual and Langaker’s definition of pregnancy, missed miscarriages would not count as *false* positives, for there is no requirement that the embryo or fetus is continuing to develop, merely that it is implanted.) Given all this, we might say that pregnancy is objectively verifiable to a high, though not absolute, degree of accuracy.

For Smajdor and Räsänen, objective verifiability also entails, however, that whether or not one is pregnant “does not depend on how the person feels about it, unlike infertility, into the definition of which, the wish for a child is written” (2025, 42).^10^ There are a number of things that need to be clarified here. One, it was Kukla’s (2019) argument that values are written into the definition of infertility, and Smajdor and Räsänen claim that pregnancy is unlike this (and this presents a reason for counting pregnancy as a disease, contra infertility). While I am not sure I agree with Kukla on infertility, I rather agree with Smajdor and Räsänen that pregnancy is objectively verifiable—at least on most of the considered definitions. This should not be taken to suggest however, that the meanings of pregnancy or meanings that attend pregnancy, are not a matter of how people feel about it. And if one adopts a definition of pregnancy that requires a ‘woman’ or a person of any social identity to be the subject of it, then insofar as the meaning of ‘woman’ or of any social identity is subjective or otherwise normative (rather than experimentally measurable), this also cuts against the kind of objective verifiability that Smajdor and Räsänen have in mind.

There is one more issue in Smajdor and Räsänen’s argument which has bearing on whether pregnancy reflects a unified phenomenon. In their discussion of the health risks of pregnancy, Smajdor and Räsänen lump pregnancy together with childbirth. They describe labor as the “second stage” of pregnancy (2025, 37). They state that “[t]his second stage is far riskier than the first, in terms of long term threats to life and health” (2025, 37). Labor certainly involves extreme pain, powerful cramps, the ripping, stretching, and damaging of tissue, and is riskier than the so-called “first stage” of pregnancy, as Smajdor and Räsänen claim (2025, 37). However, out of the definitions of pregnancy canvassed above, only one even potentially includes birth: #5, under which pregnancy “begins with the fertilization of an egg and continues through to the birth of the individual.” Even here, it appears that pregnancy ends upon birth, thus birth does not seem to be included in the phenomenon, although this is ambiguous. Avise suggests that pregnancy is the phase that *precedes* birth (definition #7, emphasis mine). Furthermore, to construe birth as part of the phenomenon of pregnancy is to take a dubious, teleological view of pregnancy (Scuro 2017; Browne 2022). Many cases of pregnancy do not result in birth, but result instead in abortion (spontaneous or elective), or fetal resorption. Furthermore, Smajdor and Räsänen themselves reject teleological views of functions in their critique of dysfunction theories of disease. In this case, not only may lumping together pregnancy and birth reflect an interested conflation, it skews their comparison of the risks of pregnancy to the risks of measles qua paradigmatic example of disease.^11^ Setting these problems aside, it appears that at least for parts of their argument, Smajdor and Räsänen themselves are not discussing a unified phenomenon. Their claim that pregnancy meets the second criterion is therefore weakened.

In the end, it is not fully clear whether pregnancy meets Smajdor and Räsänen’s second criterion for disease. It does not, if all the phenomena that the disparate definitions refer to jointly, are pregnancy. It does, if we take pregnancy to refer to *one* of the unified phenomena stated above, although the choice of referent would have to be justified. But it does not, if we take birth to be part of the phenomenon of pregnancy, as Smajdor and Räsänen suggest it is. Overall, it appears that while ‘pregnancy’ *could* refer to a unified phenomenon, Smajdor and Räsänen’s assumption that it does is premature.

### Criterion 3: medical risks

Smajdor and Räsänen’s third suggested criterion for pregnancy being a disease was that its risks were medical rather than social: “The risks of pregnancy are *medical* to use Kukla’s terminology, rather than social, though of course they can be, and often are, both” (2025, 42).^12^ Many complications arise here, having to do with defining ‘risk,’ as well as with the nature of what is ‘medical’. Defining risk is like defining disease: risk is not a purely neutral matter of chance or probability (a “Boorsean” view of risk); it also includes a normative factor—perhaps harm or undesirability (Peschard et al. 2022, 5–6; Hansson 2023).^13^ Thus, the same problems attend conceptions of risk as attend any issue where disagreement occurs about what is harmful or undesirable: risk is subjective, its definition susceptible to bias by various social values. And Smajdor and Räsänen take this issue seriously, as evidenced by their discussion of Cooper’s subjectivist theory of disease.

Setting aside the definition of risk, we can talk about it intuitively for the time being. With regard to what is ‘medical’, it should be noted that Kukla uses the term intuitively to refer to whatever effects of infertility contrast the ‘social’ (Kukla 2019). And while it seems to me that medicine is itself a social practice, rendering the contrast ambiguous, the term is meant to refer to practices that study or intervene in bodies physiologically. So, presumably the medical risks of pregnancy, as they are discussed by Smajdor and Räsänen, include its likely or potential negative physiological effects. These are what Smajdor and Räsänen discuss when they compare pregnancy to measles. The physiological risks of pregnancy they cite include

back pain, bleeding gums, headaches, heartburn and indigestion, leaking from the nipples, nosebleeds, pelvic pain, piles, stomach pain, stretch marks, swollen ankles, feet and fingers, tiredness and sleep problems, thrush, vaginal bleeding, vaginal discharge, vomiting, morning sickness and weight gain. (Smajdor and Räsänen 2025, 37)

Childbirth or labor involves “extreme pain, powerful cramps, and the ripping, stretching, and damaging of tissue” (Smajdor and Räsänen 2025, 37). Again, they note that the “second stage” of pregnancy is far riskier than the first. Furthermore, pregnancy carries with it the very serious risk of mortality. Smajdor and Räsänen note that “If medical services are not available, many pregnant people will be seriously injured as a result of second-stage pregnancy, and a significant number of them will die” (Smajdor and Räsänen 2025, 38). While it is not clear to me that childbirth or labor are parts of the phenomenon of pregnancy (as discussed with regard to the second criterion), I will grant this for the purpose of considering whether pregnancy meets the third criterion, as Smajdor and Räsänen argue.^14^

One problem here is that it is not clear what factors contribute to certain risks associated with pregnancy. Admittedly, some risks of pregnancy presumably arise primarily as a matter of pregnancy’s physiological effects, for instance, thrush or morning sickness. But many risks are a result of the social determinants of health, including childbirth-related maternal mortality (Maness and Buhi 2016; Wang et al. 2020; Backes and Scrimshaw 2020; Luby et al. 2023). As Smajdor and Räsänen themselves claim, injury and death are likely to occur *if medical services are not available*. In the United States, the CDC has found that four out of five pregnancy-related deaths were preventable (CDC 2024). In that case, is it pregnancy or the absence of appropriate healthcare that constitutes a risk of injury or death? It is likely that such factors can never be fully disentangled. We can conduct no (ethical) experiments on human pregnancy and birth that control for the presence of healthcare. But we can make the plausible inference that many risks associated with pregnancy are social by comparing the effects of pregnancies that occur in the presence and the absence of healthcare. The World Health Association found that in 2020 the maternal mortality rate in high income countries was just over 3% of that found in low income countries (Lale Say et al. 2014). Indeed, maternal mortality rates can decrease with improved healthcare (Office of the Surgeon General (OSG) 2020; Howell and Zeitlin 2017). If a lack of healthcare were not a factor in maternal mortality, it would not make sense to include reductions in maternal mortality as one of the Millenium Development Goals (World Health Organization 2018). This suggests that social factors explain a much larger proportion of the risk of pregnancy. Still, there may be a risk “floor” that pregnancy represents—a baseline level of risk that is higher than the level for non-pregnant individuals. This is because it is arguably a physiological state that makes one more susceptible to common health risks of the human population in general. We can safely assume that *both* pregnancy *per* *se* and the lack of healthcare contribute to injury or death associated with pregnancy. Nevertheless, it appears that the risks of pregnancy are primarily social, and by a large margin.

Furthermore, there appear to be a great number of other social risks associated with pregnancy. One is workplace discrimination. Globally, 20 (out of 165) countries have no legal prohibitions on pregnancy discrimination, and at least 82 do not guarantee the right to return to work after pregnancy (in a comparable work position to the one held prior to pregnancy) (Addati, Cassirer, and Gilchrist 2014, 73ff). More than half of women globally work in the informal sector, thus even if their countries have legal protections against pregnancy discrimination, these do not apply to such workers in practice (Kamande 2024). Needless to say, the loss of a job, or the failure to secure a job, is a significant social risk, particularly in pregnancy. In the United States, women (and those who may be pregnant who do not identify as women) continue to face employer discrimination despite the fact that it is illegal (McCann and Tomaskovic-Devey 2021). Pregnancy discrimination is widespread, yet there are many barriers to establishing the prevalence and the particular instances of discrimination. For instance, data may rest on the number of litigated cases, but not all pregnancy discrimination reports or cases reach litigation (McCann and Tomaskovic-Devey 2021). Furthermore, pregnancy discrimination can occur during recruitment or hiring (Addati et al. 2014), which means those who are pregnant may never know or be able to prove that they were discriminated against. However, the data that do exist do not tend to define pregnancy discrimination, nor disambiguate it from, e.g., maternity-related discrimination. Thus, the extent of discrimination which is linked specifically to pregnancy (under some definition) is hard to establish. Despite these ambiguities, it is safe to say that there are significant, and perhaps life-altering, employment-related social risks associated with pregnancy.

Another apparent social risk associated with pregnancy is gender- or sex-based violence. Here, too, the relationship between pregnancy and violence is hard to establish. However, homicide is the leading cause of death in pregnancy in the United States (Campbell et al. 2021). Homicide is considered a “pregnancy-associated” cause of death, however, which means it is not taken to be caused by pregnancy *per* *se* (Campbell et al. 2021, 236). In this case, it would take further conceptual work (on ‘pregnancy’, on ‘risk’, as well as on the criteria for association, causal or otherwise) to establish that homicide is a risk of pregnancy. But this is likewise the case for the ‘medical’ risks of pregnancy cited by Smajdor and Räsänen. For instance, they include labor as a stage of pregnancy, when labor may not be a stage of pregnancy, and that they include symptoms such as stretch marks as risks when these are only ‘risks’ if they are evaluated negatively (more on this below). Still, in the data driving the finding on homicide, pregnancy-associated deaths include those deaths that occur up to a year postpartum. In this case, how we should weight homicide as a social risk of pregnancy, is debatable. Nevertheless, it appears that pregnancy increases the risk of homicide relative to the risk for non-pregnant women (Wallace et al. 2016).

For those who do not wish to become parents, pregnancy also poses the risk of parenthood. This is a fraught point, complicated by the lack of a definition of risk. If the definition of risk involves any subjective normative factors (which, like disease, it seemingly must, lest it merely reflect neutral probabilities), then assuming that parenthood is a social category, for those who aim to avoid parenthood pregnancy distinctly carries such a risk.^15^ There may also be subtler social risks of pregnancy: social surveillance and pressures, difficulties managing new relationship dynamics, forms of (often racialized) sexism or misogyny, or mundane externalities associated with scheduling, attending, and paying for a great number of prenatal appointments or classes. There are doubtless many more such risks, and I cannot enumerate them all or evidence their prevalence empirically here. This discussion should suffice to show, however, that it is far from clear whether the risks of pregnancy are primarily medical.

Again, how to weigh these social risks relative to medical risks would require further, detailed conceptual and empirical investigation. As with the social risks related to pregnancy, it would be important to investigate how the medical risks related to pregnancy pertain to pregnancy per se, including by selecting a consistent definition of pregnancy as a variable to be associated with its various ‘risks’. It would also be necessary to select a definition of risk. In the end, Smajdor and Räsänen have not established that the risks of pregnancy are primarily medical.

### Criterion 4: Social-Value independence

The fourth criterion for pregnancy being a disease is that its ‘risks’ must not only be risks within a particular set of social values. What this means is that the ‘risks’ of pregnancy must be risks regardless of the particular set of social values present for pregnancy to count as disease. The apparent value in question here is pronatalism, which Smajdor and Räsänen claim may prevent us from recognizing pregnancy as a disease (2025, 38–39, 42). Thus, to meet the fourth criterion for disease, pregnancy must carry risks whether pronatalist values are present or not. And Smajdor and Räsänen suggest that pregnancy does carry such risks on the grounds that the probable physiological consequences of pregnancy would occur regardless of the presence or absence of pronatalism.

However, for the probable physiological consequences of any condition to count as risks requires commitment to some particular set of social values. If this were not the case, then it is hard to see how those consequences could count as harmful, undesirable, or otherwise bad. Again, ‘risk’ is only risk if it includes a normative component such as a judgment of harm or undesirability, otherwise it would be merely a neutral matter of chance or probability (Peschard et al. 2022, 5–6; Hansson 2023).^16^ In this case, the required normative component would seem to be the social value that tells for the harmfulness, undesirability, or badness of injury and pain, as may occur with many physiological conditions. Thus, strictly speaking, for the probable physiological consequences of pregnancy to count as risks, they must count as such even in the absence of the social values that allow us to judge injury and pain as harmful, undesirable, or otherwise bad. But this creates a problem for Smajdor and Räsänen since they have argued that theories of disease that depend on a particular sets of social values should be distrusted.

One way to resolve this inconsistency is to point out that the social values required to count the probable injury and pain of conditions such as measles (a paradigmatic disease) as well as of pregnancy as harmful, undesirable, or otherwise bad, and thus as risks, are rather well-established, fairly universal, and justified. One could argue that, in contrast, the social value of pronatalism is not. Smajdor and Räsänen may have had in mind some subset of parochial or not well-justified social values as the kinds of values that the risks of pregnancy must be independent of, rather than taking them to be independent of values we may consider well-established, fairly universal, and justified. But not only is it controversial which kinds of values we should consider to be well-established, universal, and justified and which we should not, it is not clear that this is what Smajdor and Räsänen actually had in mind.

Smajdor and Räsänen rejected views of disease that both relied and did not rely on social values in general (see Section II), so it is not clear why they would accept a view of risk that either relies or does not rely on any social values. For instance, they suggest that a problem with Cooper’s subjectivist view of disease is that it relies on “subjective value” (2025, 38).^17^ They write that, “if values play a role in classifying disease, we should not expect this to yield a neat, reliable distinction between what constitutes a disease and what does not” (2025, 38). But the same can be said of risk: without social values, or a particular set of social values, we should not expect to have a neat, reliable distinction between what constitutes a risk and what does not. This is not least because, as Smajdor and Räsänen note, some people value experiences or conditions that are bad for them (2025, 38).

Smajdor and Räsänen consider descriptive theories of disease, on which a phenomenon counts as disease when it is treated as such by the medical community. A descriptive account would take care of the problem of value-dependence. They claimed, however, that on descriptive accounts pregnancy counted as both a disease and not a disease, and they rejected such accounts because of this contradiction. Analogously, on such accounts of risk, certain effects of pregnancy would both be risks and not risks (as argued above). Thus, for consistency, they must reject them. In this case, they still face the problem of value-dependence.

Smajdor and Räsänen then considered dysfunction theories of disease, but they rejected them because these required value judgments (about how bodies should behave). The broad challenge Smajdor and Räsänen posed for dysfunction theories of disease regarded whose or which values should guide what we see as functions versus dysfunctions. They also criticized deriving an ‘ought’ from an ‘is’ (2025, 40). They claimed that “[t]his leap from the descriptive to the normative is enormously problematic,” and that the problem was “insurmountable” (2025, 40). Yet the same questions arise for risk, for again, without a normative component, risk refers to mere probability.

Smajdor and Räsänen also rejected Boorse’s biostatistical theory of disease. If Boorse’s biostatistical theory is interpreted as a non-normative theory of disease, then the same problem occurs that came up for descriptive theories of disease. If it is interpreted in light of Boorse’s comments on contributions to survival and reproduction, in which case pregnancy is not a disease given that it contributes to reproduction, then by the same rationale, some ‘risks’ are not risks if by undertaking them we meet some important aim. Smajdor and Räsänen also pointed out that whether pregnancy is a disease is environment-relative on a Boorsean view, since whether something contributes to survival or reproduction is environment-relative. If, for instance, full ectogestation became a reality and its use became widespread, then pregnancy would no longer contribute to reproduction. In that case, on a Boorsean view, it would count as a disease. But the same case can be made for risks: they are context-relative, as I argued in the previous section about the social determinants of health. If Smajdor and Räsänen wish to be consistent with their arguments regarding disease, they must reject all of these views of risk, for the very reasons they give for rejecting theories of disease. If they do that however, then pregnancy cannot meet the fourth criterion.

## Conclusion

Smajdor and Räsänen have not effectively made the case that pregnancy performs well on the criteria they suggest. They have shown neither that pregnancy should be classified as a disease, nor that there are pragmatic reasons for classifying it as such. To establish this would require a definition of the phenomenon they wish to classify (pregnancy) and a specification of the category into which they aim to fit it (disease), as well as a meaningful defense of how the phenomenon fits the category. These features are missing from their account.

The search for a satisfactory theory of disease is ongoing. In addition to integrating broadly naturalist with broadly normativist approaches to theorizing disease, a theory may need to include contingent historical factors that determine the nature of medical practices at particular times and places (see Rosenberg 1989). Setting such overarching questions aside, the present critique is motivated in part by an unfortunate implication of Smajdor and Räsänen’s argument: that on average, female bodies are more diseased than are male bodies. This is both sexist and implausible. Relatedly, Teresa Baron’s response to Smajdor and Räsänen argues that their account pathologizes women (Baron 2025). I am in broad agreement with Baron’s point; if we must include social values as part of the theory of disease (and I believe we must) then I suggest we include sex- or gender-neutrality. In making the present critique, however, I have avoided arguing from unacceptable conclusions, since what counts as unacceptable is contestable and varies greatly among readers. Instead, I show that whether the feminist critique is compelling to the reader or not, Smajdor and Räsänen’s argument does not succeed by its own criteria.

## Data Availability

No datasets were generated or analysed during the current study.

## Footnotes

1 Quill Kukla was writing as Rebecca Kukla in their (2019).

2 See Colgrove and Rodger (2025) and Rezkalla and Smith (2025) for evaluations of this argument.

3 See (Baron 2025) for a critique of this argument.

4 That it is value-free has been disputed by Kingma (2007).

5 Interestingly, this would also mean that whether pregnancy is a disease or not would likely be species-relative.

6 Notably, this issue is not limited to the biomedical context. All empirical inquiry depends upon the prior selection—implicit or explicit—of independent and dependent variables. Another way to put this point is that the results of empirical inquiry depend upon how the target system(s) with which it is concerned are demarcated (see Elliott-Graves 2020).

7 The searches were conducted using Google. The search for “medical definition of pregnancy” included the first two definitions enumerated in this section within the first three results. The search for “biological definition of pregnancy” included definition (2) (cross-listed in the results for the “medical definition of pregnancy”) as the fourth result, definition (3) as the fifteenth result, definition (4) as the fifth result, definition (5) as the ninth result, and definition (6) as the first result. While legal contexts may also provide definitions, no legal definitions of pregnancy are presented here because the context of the central question is squarely biomedical.

8 Some results returned by the searches did not offer any definition and thus were not included. Other sources were not included if the definitions they offered were close analogues of those presented below.

9 Avise notes that, conventionally, parturition is called “birth” or “delivery” in viviparous species, while it is called “extrusion” or “oviposition” in egg-laying species (2013, 39).

10 Contra Kukla as well as Smajdor and Räsänen, I suggest it is not necessarily the case that the wish for a child is written into the definition of infertility. Some people may be relieved to be infertile, may have no clear reaction to infertility, or simply be unaware of being infertile, and be no less infertile. While many studies show distress at infertility diagnoses (e.g., Boivin et al. 2023), suggesting that those who are infertile do wish for a child, the sample populations surveyed in such studies are wholly non-random since they reflect the attitudes only of those who have sought medical diagnosis and intervention to aid with their goal of biological reproduction. This grossly biases the results of such studies, and casts doubt on the claim that the wish for a child is written into the definition of infertility.

11 Setting aside resorption, there does not appear to be reliable data on the relative safety of spontaneous or elective abortion relative to childbirth. One often cited study by Elizabeth Raymond and David Grimes shows that childbirth is fourteen times more risky than elective abortion (2012). But Byron Calhoun pointed out significant methodological problems with assessing these risks (2015), so the result is in question. Another study lumped together mortality associated with spontaneous and elective abortion, as well as with ectopic pregnancies, along with other causes such as suicide, homicide, accidents, and other “natural causes” (Reardon and Thorp 2017). Regardless of the relative risks of phenomena that end pregnancy other than birth, it is not clear that the phenomena that end pregnancy are part of the phenomenon of pregnancy *per* *se*.

12 Smajdor and Räsänen (as well as Kukla) treat ‘the medical’ and ‘the social’ as *exclusive* disjuncts, not inclusive ones. This is because they take this distinction to differentiate between disease and non-disease. Thus, strictly speaking, Smajdor’s and Räsänen’s claim that “The risks of pregnancy are *medical* to use Kukla’s terminology, rather than social, though of course they can be, and often are, both” (2025, 42), is contradictory. Were ‘the medical’ and ‘the social’ inclusive disjuncts, the dialectic of both arguments would collapse. Thus, I interpret Smajdor and Räsänen to hold that the risks of pregnancy are *mostly* medical. This interpretation aims at a middle ground between their claim that the risks of pregnancy are medical rather than social and their claim that the risks of pregnancy are both medical and social.

13 Defining risk as mere ‘chance’ in this context would necessitate the inclusion of the positive physiological effects of pregnancy. I set these aside, although Smajdor and Räsänen acknowledge them (2025, 37).

14 It is worth noting, however, that the risks under discussion are risks only of human pregnancy. Thus, if one defines pregnancy as a phenomenon that pertains to non-human as well as to human animals, then the risks may need to be disambiguated by species. In that case, however, the risks of human pregnancy will differ from the risks of pregnancy.

15 It is true that parenthood can be understood in many ways. One understanding of parenthood is as a social role. Another understanding of parenthood is as a matter of genetic descent. It is beyond the scope of this argument to establish the extension or meaning of ‘parent’, but I suggest it is plausible to understand parenthood socially here for the purpose of claiming that it is the social role that some take to be a risk of pregnancy.

16 Alternatively, if it carried a judgment of benefit or desirability, then it would be luck (or the like), not risk. This is borne out by the fact that Smajdor and Räsänen count some probable physiological consequence of pregnancy, such reductions of arthritis symptoms or reduction in incidence of breast cancer, not as risks but as benefits (2025, 37).

17 I am setting aside the distinction between subjective value and social value here, as do Smajdor and Räsänen.
